# Flip-Flopping Retinal in Microbial Rhodopsins as a Template for a Farnesyl/Prenyl Flip-Flop Model in Eukaryote GPCRs

**DOI:** 10.3389/fnins.2019.00465

**Published:** 2019-05-07

**Authors:** Arnold De Loof, Liliane Schoofs

**Affiliations:** Functional Genomics and Proteomics Group, Department of Biology, Zoological Institute, KU Leuven, Leuven, Belgium

**Keywords:** GPCR activation, farnesol, mevalonate pathway, G protein, prenylation, juvenile hormone, allostery cryo-EM

## Abstract

Thirty years after the first description and modeling of G protein coupled receptors (GPCRs), information about their mode of action is still limited. One of the questions that is hard to answer is: how do the allosteric changes in the GPCR induced by, e.g., ligand binding in the end activate a G protein-dependent intracellular pathway (e.g., via the cAMP or the phosphatidylinositol signal pathways). Another question relates to the role of prenylation of G proteins. Today’s “consensus model” states that protein prenylation is required for the assembly of GPCR-G protein complexes. Although it is well-known that protein prenylation is the *covalent* addition of a farnesyl- or geranylgeranyl moiety to the C terminus of specific proteins, e.g., α or γ G protein, the reason for this strong covalent binding remains enigmatic. The arguments for a fundamental role for prenylation of G proteins other than just being a hydrophobic linker, are gradually accumulating. We uncovered a *dilemma* that at first glance may be considered physiologically irrelevant, however, it may cause a true change in paradigm. The consensus model suggests that the only functional role of prenylation is to link the G protein to the receptor. Does the isoprenoid nature of the prenyl group and its exact site of attachment somehow matter? Or, are there valid arguments favoring the alternative possibility that a key role of the G protein is to guide the covalently attached prenyl group to – and it hold it in – a very specific location in between specific helices of the receptor? Our model says that the farnesyl/prenyl group – aided by its covalent attachment to a G protein -might function in GPCRs as a horseshoe-shaped flexible (and perhaps flip-flopping) hydrophobic valve for restricting (though not fully inhibiting) the untimely passage of Ca^2+^, like retinal does for the passage of H^+^ in microbial rhodopsins that are ancestral to many GPCRs.

## Introduction

There must be a cell-physiological necessity why many ligands use a complex GPCR, of which the folded protein chain passes through the cell membrane seven times (7 TM receptors) for signaling. Theoretically, they could instead use a single transmembrane protein helix with a ligand binding site located on a “flag” extending into the extracellular environment and a stretch ending in the cytoplasm to which a G protein (complex) can be attached/associated.

This would be the easiest and energetically cheapest way for transducing allosteric changes in the receptor induced by ligand binding. The fact that instead a much less energy-friendly multi-helix bundle complex with (at least) 7 TMs made it in evolution, is a compelling argument that, perhaps, it is not the allosteric change in one or several of the helices itself that is important. An alternative explanation is that a bundle of transmembrane protein helices enables the formation of an intramolecular microchannel for a signaling ion, particularly for H^+^ as in rhodopsins, and for Ca^2+^ as in rhodopsin-descendants (the modern GPCRs). A local and transient change in the concentration of the ion involved then initiates the signaling cascade. This consideration/argument raises the question about the advantages of a ligand binding pocket in an intramolecular microchannel, and about the mechanism, which in unstimulated cells minimizes the untimely gating of this microchannel. We hypothesize that the prenyl-group of G proteins plays a prime role in microchannel gating. Briefly, the model we propose states that prenylation serves the function of an installed hydrophobic flexible molecular valve to restrict the untimely influx of Ca^2+^, analogous to a cork into a bottle neck. The mechanism we propose is neither in conflict with the “consensus model” nor with recent detailed molecular models for GPCR activation obtained with solid-state NMR (Kimata et al., 2015) and Cryo-EM (Liang et al., 2017, 2018; Kang et al., 2018; Safdari et al., 2018). These methods require the use of wetting agents/detergents for sample preparation, and such treatment will disturb attachment of the prenyl group, even if it withstands the treatment at all. Wigglesworth (1969) reported that the use of wetting agents abolished the activities of farnesol and its juvenile hormone (JH) esters in bioassays.

## A Short Introduction to Ca^2+^ Homeostasis. The Mevalonate Pathway

Calcium is better known for its beneficial effects, e.g., as a secondary messenger in signaling pathways, as well as from its most visible role in the manufacture of the skeleton in corals, molluscs, crustaceans, vertebrates, etc., rather than for its toxicity. Yet, Ca^2+^ is the most abundant toxin on earth, and it can act as a secondary messenger *because it is toxic* (De Loof, 2015, 2017). This is due to the fact that changes in Ca^2+^ concentrations have profound effects on the 3D conformation of various macromolecules, and changes their functionalities as a result. Particularly, the conformation-influencing effect of changing Ca^2+^ concentrations on the proteinaceous contractile apparatus of muscle cells, as well as Ca^2+^-induced changes in chromatin configuration (Lai et al., 2009) are clear examples.

**FIGURE 1:**
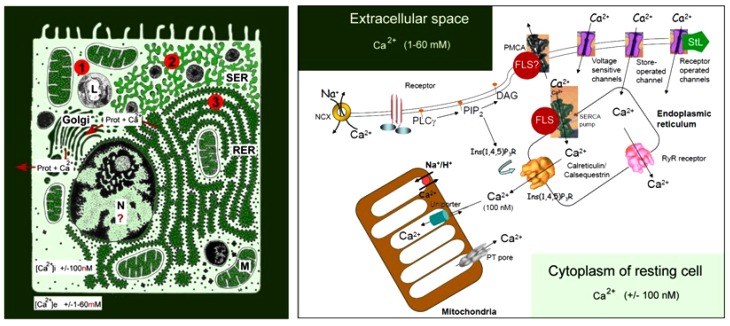
Schematic representation of the main Ca^2+^ gradients in eukaryotic animal cells (Left panel) and of the main players in Ca^2+^-homeostasis (Right panel). Left: schematic representation of the Ca^2+^ gradient (adapted from De Loof, 2015, 2017). The different shades of green are not meant to give an exact representation of differences in Ca^2+^-concentration. L, lysosome; N, nucleus; M, mitochondrion; RER, rough endoplasmic reticulum; SER, smooth endoplasmic reticulum. The red dots with 1, 2, and 3 correspond to the main mechanisms for keeping [Ca^2+^]i low. (1) Little influx of Ca^2+^ through the plasma membrane that can be countered by the activity of Ca^2+^-ATPases in the plasma membrane (PMCAs); (2) more influx and role for temporary storage of Ca^2+^ in membrane-limited organelles, in particular the SER; (3) high influx of Ca^2+^ triggers the removal of excess Ca^2+^ through the secretion of Ca^2+^-binding/transporting proteins via the RER. From De Loof (2017). Right: the major events in the Ca^2+^-homeostasis system (slightly modified after Orrenius et al., 2003). The long legend as originally formulated by Orrenius et al. (2003) is not repeated here De Loof (2017).

The huge concentration gradient of Ca^2+^ over the plasma membrane, which is about 20,000-fold higher in blood (2 mmol Ca^2+^) (Figure 1) compared with the cytoplasm of unstimulated (resting) cells “drives” Ca^2+^ into cells at any time that Ca^2+^ gates open up.

The lipid bilayer of biomembranes is impermeable to Ca^2+^, but many complex proteinaceous transmembrane proteins permit the passage of Ca^2+^ when properly stimulated to form a transient intramolecular microchannel. Examples are the well-documented types of canonical Ca^2+^ channels. Excess [Ca^2+^]i, exceeding 100 nmol, that entered the cell has to be pumped out of the cytoplasm, quickly and efficiently, by Ca^2+^ pumps located in the plasma membrane, known as Plasma Membrane Ca^2+^ ATPases or PMCAs, and/or by Ca^2+^ pumps in some of the intracellular membrane systems, such as the abundantly present SERCA Ca^2+^ pump (SR Ca^2+^-ATPase) in myocytes (for figures see Orrenius et al., 2003; De Loof, 2015, 2017).

G protein coupled receptors are only one of the participants in the Ca^2+^ homeostasis system. Although the amount of Ca^2+^ that -upon their activation by, e.g., ligand binding- can pass through intramolecular channels is low, the well-documented process of “Ca^2+^-induced Ca^2+^ release” can cause substantial local shifts in [Ca^2+^]i. Pump- and channel activity thus have to be kept in balance on a continuous basis. This requires a finely tuned coordination between all elements that influence Ca^2+^-influx and efflux. Indeed, it does not make sense to activate a PMCA, and simultaneously open Ca^2+^-channels in the plasma membrane. Hence, the different elements involved in Ca^2+^-homeostasis must have been in place and started functioning very early in evolution, enabling the ancestral cells to avoid Ca^2+^-induced cell death. During the next billions of years of evolution, the Ca^2+^homeostasis system has been shaped to near perfection. Ca^2+^ channel- and pump types have been remarkably well-conserved in evolution.

**FIGURE 2:**
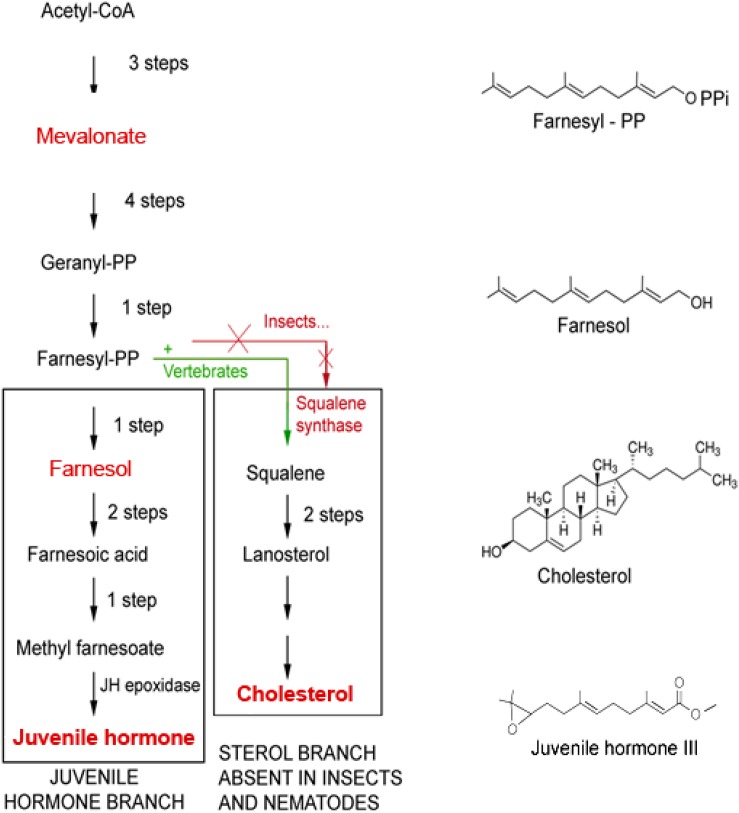
The mevalonate biosynthetic pathway in which farnesol is formed. The initial steps up to the synthesis of farnesyl-PP and farnesol are identical in insects and vertebrates. Later steps are different. Because Ecdysozoa ( = arthropods and nematodes) do not have the gene coding for squalene synthase, they cannot synthesize squalene and cholesterol by themselves. They acquire cholesterol from the food, as a vitamin, or by metabolization of some (ecdy)steroids. Insects can make esters of farnesol, the Juvenile Hormones (JHs), that are more potent than farnesol itself in specific bioassays for JH. Vertebrates do not make JHs, but some plants do. Adapted from Bellés et al. (2005) and De Loof et al. (2014).

The mevalonate biosynthetic pathway (Figure 2), with farnesol as a key intermediate, functions as a precursor of JH(s) in insects is also evolutionarily very ancient (De Loof and Schoofs, 2019). Interestingly, the mevalonate pathway also displays a prominent role in Ca^2+^ homeostasis (this paper).

## Why Is Farnesol Such a “Noble Unknown” in (Mainly Vertebrate) Physiology and Endocrinology?

The reason why farnesyl- as a molecular valve with a role in restricting untimely Ca^2+^-influx into the cytoplasm model has not been formulated before by other researchers, is that some of the experimental data supporting this model were published half a century ago, thus long before research on GPCRs emerged. At that time, no attention was given to the possible functional importance of farnesol’s high molecular flexibility as indicated by its Rotatable Bond Count of 7 (PubChem: *trans, trans*-Farnesol), in combination with its horseshoe-shape (see later and Figure 6) and hydrophobicity. In addition, it was assumed that farnesol is neither a hormone, nor an “inbrome” (De Loof et al., 2015). Instead, the general view was that farnesylpyrophosphate only serves a role as a precursor for squalene in the mevalonate pathway, and that farnesol itself, if it would occur at all, has no specific function. That farnesol can have a role in itself in Ca^2+^ homeostasis has, however, been convincingly demonstrated by the electrophysiologists Luft et al. (1999) and Roullet et al. (1999).

**FIGURE 3:**
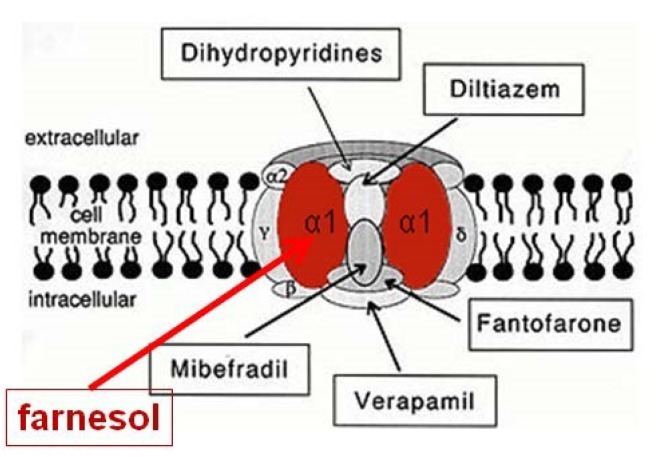
Farnesol is an inhibitor of some types of voltage-gated Ca^2+^ channels. Luft et al. (1999) and Roullet et al. (1999) demonstrated for the first time that farnesol binds to the α1 subunit, which is the pore forming unit of voltage-gated Ca^2+^ channels. Ligands for the other subunits are well-documented (Figure modified after Wikipedia, 2018a). Borrowed from De Loof and Schoofs (2019) (Open access).

They showed that the endogenous sesquiterpenoid farnesol (the *trans–trans* isomer) is a potent inhibitor of voltage-gated Ca^2+^-channels in rodents and humans (Figure 3); other types of organisms have not yet been tested in this respect. Although these authors assumed that farnesol acts from inside the cells as an inbrome and not as a hormone, a hormonal role for farnesol has been demonstrated in insects. When tested in bioassays that monitor activity of JHs, which are esters of farnesol (for figure, see De Loof et al., 2014), it has been shown that many farnesol-like substances (FLSs), in particular the JHs, are more active than farnesol, some even several orders of magnitude (Wigglesworth, 1969). Thus a hormonal role for farnesol cannot be excluded. However, the evolutionarily ancient role of farnesol/FLS was certainly not a hormonal one, because the mevalonate pathway with farnesol as one of its intermediates was most likely already present in unicellular eukaryote ancestors of all animals, as it also occurs in the choanoflagellate Opisthokonts (Cavalier-Smith, 2017; De Loof and Schoofs, 2019). The high degree of evolutionary conservation of both the mevalonate pathway and the Ca^2+^-voltage-gated channels suggests that the mode of action of farnesol underlying its role in Ca^2+^-homeostasis, may be universal in all eukaryotes.

Through another approach, De Loof (2015) and De Loof et al. (2015) advanced the hypothesis that farnesol may be the natural cognate ligand of the SERCA-Ca^2+^ pump, which is displaced by the SERCA pump blocker thapsigargin. Thapsigargin raises the cytosolic Ca^2+^ concentration by blocking the ability of the cell to pump calcium into the lumen of the sarcoplasmic and endoplasmic reticula (SER and RER). Like farnesol, the plant toxin thapsigargin, is also a sesquiterpenoid. It induces apoptosis like absence of JH does during metamorphosis of holometabolous insects (De Loof et al., 2014).

Store depletion can secondarily activate plasma membrane Ca^2+^ channels allowing an influx of calcium into the cytosol (Rogers et al., 1995; Wikipedia, 2018g). Whether the inhibitory effect of farnesol on voltage-gated Ca^2+^ channels also applies to the Ca^2+^-channels present in the SER and RER has, to our knowledge, never been investigated. During metamorphosis of holometabolous insects the well-documented drop to zero of the titre of endogenous sesquiterpenoids, farnesol and/or JHs, causes programmed cell death/apoptosis in particular in those cell types with a very well-developed RER (De Loof, 2015, 2017). This suggests that the luminal gradient of Ca^2+^ in the SER/RER collapses due to the opening of Ca^2+^ channels. Ca^2+^-induced apoptosis will result (Orrenius et al., 2003). Other mechanisms have since been suggested, e.g., Kowluru (2017) and Brooks et al. (2018).

## A Non-Hormonal Prenylation Activity of Farnesol/FLS

Farnesol/FLS with a role as a hormone like in insects starts acting at the extracellular side of cells, at the contact site between the blood and the plasma membrane. Next it may diffuse into the intracellular membrane system (De Loof et al., 2014; De Loof, 2015, 2017). Yet, there is an equally important other possible mechanism of action, namely at the border between the cytoplasm and the plasma membrane with its numerous embedded helix bundle transmembrane proteins, in particular the GPCRs and their associated G-proteins. Here prenylation is the mechanism involved. Indeed, farnesyl- that is intracellularly synthesized in the mevalonate pathway, also has non-hormonal activity, as illustrated by its role in Ca^2+^-homeostasis (§2). GPCRs are key cell-surface proteins that transduce external environmental cues into biochemical signals across the membrane (Thal et al., 2018). They are intrinsically allosteric proteins that interact via spatially distinct yet conformationally linked domains with both endogenous and exogenous proteins, nutrients, metabolites, hormones, small molecules, and biological agents (Bondke Persson, 2013). This explains why they play such an important role in cell physiology and in endocrinology. Yet, their possible link with the mevalonate pathway is seldom mentioned in the literature.

This paper advances arguments in favor of the view that such link may help to clarify how allosteric changes in a GPCR may finally result in activation of the two possible downstream pathways (see later and Figure 5). The influx of relatively larger amounts of Ca^2+^ through canonical Ca^2+^ channels is a major event, with important physiological impact. However, in addition to such Ca^2+^ channels, there are also numerous transmembrane proteins in which an intramolecular microchannel exists, that upon being stretched by, e.g., ligand binding-dependent allosteric changes, allows some Ca^2+^ or/and H^+^ to enter the cytoplasm. Thus, in order to keep [Ca^2+^]i low, such micro-channels must also be kept closed as much as possible.

## From Microbial Rhodopsins to Eukaryotic GPCRs. Retinal

**FIGURE 4:**
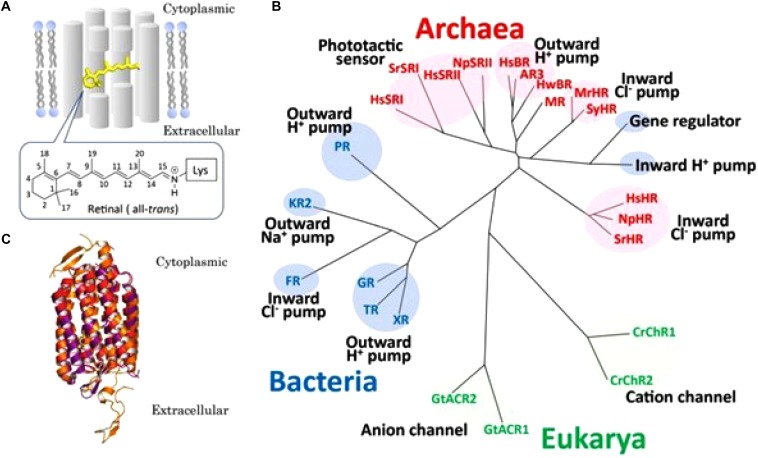
Overview of microbial rhodopsins. **(A)** Schematic drawing of a seven-transmembrane microbial rhodopsin, where an all-*trans* retinyl chromophore (yellow) is covalently tethered to a specific Lys residue of the apoprotein opsin via a protonated Schiffbase linkage. Numbers on the retinal represent the cognate carbon atoms. **(B)** Phylogenetic tree of microbial rhodopsins constructed by ClustalW software program. Microbial rhodopsins are widely distributed throughout all domains of organisms, bacteria (blue), Eukarya (green) and Archaea (red), with a wide variety of biological functions (pumps, channels, and sensors). See text in original paper by Kaneko et al. (2017) for the abbreviations of the names of microbial rhodopsins. **(C)** Superposition of crystal structures of *Halobacterium salinarum* bacteriorhodopsin (purple) (HsBR; PBD ID1C3W), *Natronomonus pharaonis* sensory rhodopsin II (red) (NpSRII; PBD ID 1JGJ) and chimeric cation channel rhodopsin (ChR) from *Chlamydomonas reinhardtii* ChR-1 (CrChR1) and ChR-2 (CrChR2) (orange) (C1C2; PDB ID 3UG9). The retinal chromophore is colored yellow. Figure and legend borrowed from Kaneko et al. (2017) (Figure 1), Open access.

With respect to the evolutionary emergence of GPCRs, cell-physiological archeology (Figure 4) may help to frame an important issue in the transition from prokaryotes to eukaryotes, about 2.7 billion years ago. GPCRs are only found in eukaryotes, including yeast, choanoflagellates and animals. To date they constitute a large protein family of receptors that detect molecules outside the cell and activate internal signal transduction pathways and, ultimately, cellular responses. According to Zhang et al. (2014) GPCRs may have evolved from the prokaryotic world. More specifically, about 80% of “modern” GPCRs are thought to have evolved out of ancient microbial rhodopsins (type-I rhodopsin). Some microbial rhodopsins function as H^+^ pumps, others as cation or anion channels, as Na^+^ or Cl^-^ pumps or as photosensors (Kaneko et al., 2017, from whom Figure 4 is borrowed, with copyright permission). Thus, in microbial rhodopsins intramolecular transport of inorganic ions is rather the rule than the exception. The chromophore retinal was essential for the proper functioning of microbial (prokaryotic) rhodopsins. A most instructive figure on the functional conversion of rhodopsins in the course of evolution is Figure 2 in the paper by Kaneko et al. (2017).

During the course of evolution, retinal lost its monopoly. Indeed, the number of ligands that bind and activate contemporary GPCRs include light-sensitive compounds, odors, pheromones, hormones, and neurotransmitters, that vary in size from small molecules and peptides to large proteins (Mertens et al., 2004; Caers et al., 2014). This means that the number of different GPCRs is high. Indeed, 7% of all predicted protein-coding genes in the worm *C. elegans* are GPCRs (Bargmann, 1998; Fredriksson and Schiöth, 2005). Most of them (∼1300) encode nematode-specific chemoreceptors (Frooninckx et al., 2012).

## The Main Bottleneck in Understanding GPCRs: Intrinsically Allosteric Proteins

To date, despite the progress obtained in recent high resolution structural studies (Thal et al., 2018), it is not yet fully understood how *allosteric transitions* (conformational 3D changes) caused by binding of, e.g., a ligand to the binding pocket of a GPCR finally yield a physiological effect inside the cell. Particularly, the role of prenylation remains enigmatic, despite some recent progress in diabetes research (Kowluru, 2017), and in uncovering the role of farnesylation of the transducing γ subunit as a prerequisite for its ciliary targeting (i.e., to outer segments of vertebrate rod photoreceptors) (Brooks et al., 2018).

Based on numerous experimental results, the widely accepted consensus on the general mode of action of GPCRs states that when a ligand binds to a GPCR, it causes a conformational (allosteric) change in the GPCR, which allows it to act as a guanine nucleotide exchange factor (GEF). The GPCR can then activate an associated G protein by exchanging the GDP bound to the G protein for a GTP. The G protein’s _α_ subunit, together with the bound GTP, can then dissociate from the β and γ subunits to further affect intracellular signaling proteins or target functional proteins directly depending on the α subunit type (G_α_s, G_α_i/o, G_α_q/11, G_α_12/13; Wikipedia, 2018d).

## G Proteins Are Absent in (Most) Prokaryotes but Are Necessary Companions of GPCRs in Eukaryotes

Two major classes of G proteins are well-documented (Gilman, 1987; Bastiani and Mendel, 2006; Wikipedia, 2018c). The first class functions as monomeric small GTPases (small G proteins: Rac1, Cdc42, Arf6, Rab27A; Kowluru, 2017), while the second class functions as heterotrimeric G protein(s) (Bastiani and Mendel, 2006, and many other papers). Receptor-activated G proteins are bound to the inner surface of the cell membrane. They consist of the G_α_ and the tightly associated G_βγ_ subunits. There are many classes of G_α_ subunits (Syrovatkina et al., 2016): Gs_α_ (G stimulatory), Gi_α_ (G inhibitory), Go_α_ (G other), Gq/11_α_, G_α_q, and G12/13_α_ are some examples. They behave differently in the recognition of the effector molecule, but share a similar mechanism of activation.

**FIGURE 5:**
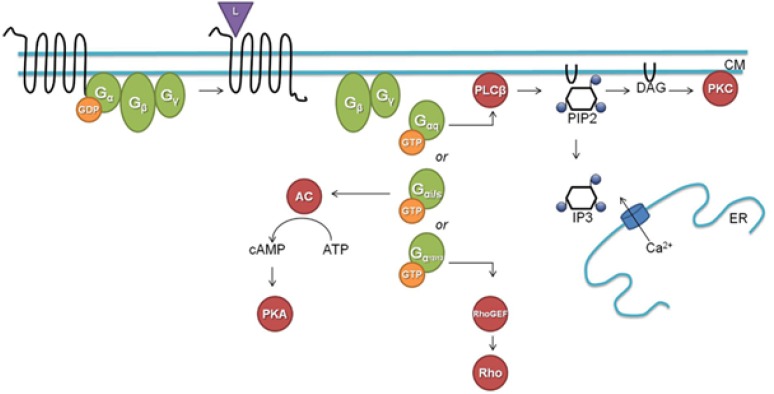
A classical downstream G protein signaling pathway (L, ligand; CM, cell membrane; GDP, guanosine diphosphate; GTP, guanosine triphosphate; AC, adenylate cyclase; cAMP, cyclic adenosine monophosphate; ATP, adenosine triphosphate; PKA, protein kinase A; PLC_β_, phospholipase C_β_; PIP2, phosphatidylinositol 4,5-bisphosphate; DAG, diacylglycerol; IP3, inositol-1,4,5-trisphosphate; ER, endoplasmatic reticulum; Rho, Rho factor; PKC, protein kinase C; GEF, guanine nucleotide exchange factor). Figure and legend borrowed from Frooninckx et al. (2012), Open access.

The classical well-documented duality in downstream signaling through GPCRs is summarized in Figure 5. Like in most other publicly available images on this topic, G proteins are shown residing closely to the cytoplasmic side of the plasma membrane. In a study on the participation of the G_α_q subunit in the 20-OH-ecdysone (20E) non-genomic pathway in larval development and metamorphosis in the insect *Helicoverpa armigera*, it was shown that before induction by 20E G_α_q was distributed throughout the cell, but that it migrated toward the plasma membrane upon 20E induction (Ren et al., 2014). This gives the impression that such translocation *after ligand binding* is part of some rescue/repair action. In this *Helicoverpa* system, G_α_q is necessary for the 20E-induced intracellular Ca^2+^ release and extracellular Ca^2+^ influx.

## Prenylation or Lipidation of G Proteins

**FIGURE 6:**
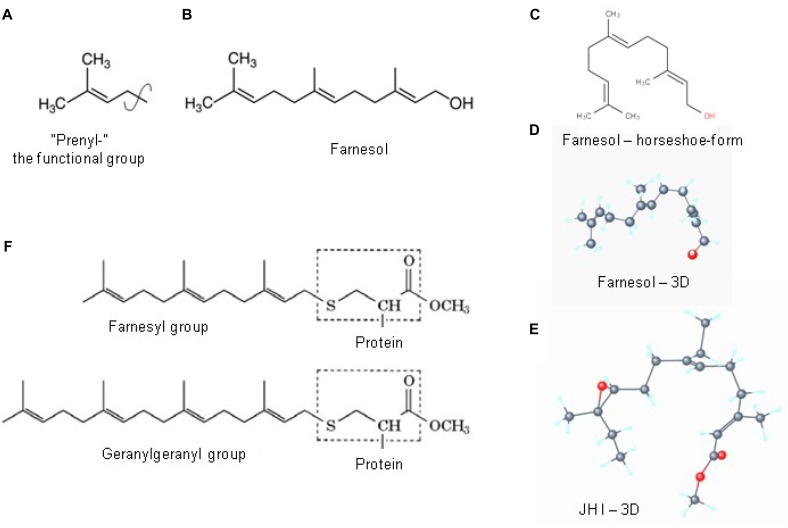
Prenylation. **(A)** The chemical structure of a “prenyl”-group and of farnesol **(B)**. In textbooks farnesol is usually depicted in its linear 2D configuration. **(C)** Its 3D conformation is horseshoe-shaped. **(D,E)** The 3D configuration showing all atoms of all-*trans* farnesol and of Juvenile Hormone I (JH I) (according to PubChem). **(F)** Protein prenylation is the covalent addition of a farnesyl- or geranyl-geranyl moiety to the C terminus of specific proteins, e.g., _α_ or _γ_ G protein. Adapted from Wikipedia (2018e,b).

**FIGURE 7:**
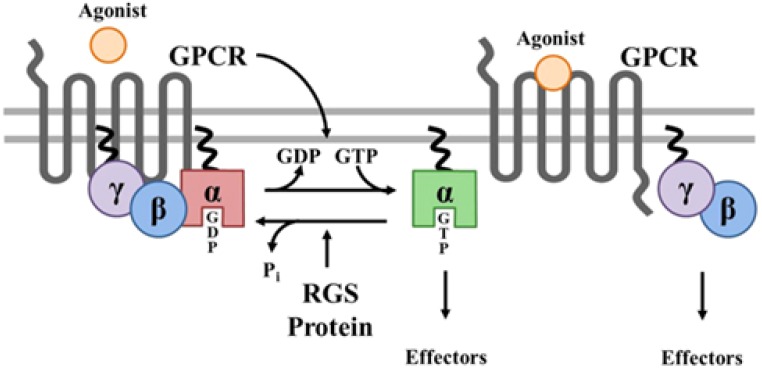
Prenylation of _α_ and _γ_ G proteins (depicted as twisted lines) and canonical regulation of GPCR signaling by RGS proteins. Agonist binding to GPCRs induces a conformational change that facilitates the exchange of GDP for GTP on the _α_ subunit of the heterotrimeric complex. Both GTP-bound G_α_ in the active form and the released G_βγ_ dimer can then go on to stimulate a number of downstream effectors. RGS proteins are GTPase accelerating proteins (GAPs) for G_α_, which function to terminate signaling through GPCRs by accelerating the intrinsic GTPase activity of G_α_ and promoting re-association of the heterotrimeric complex with the receptor at the cell membrane. Borrowed from Stewart et al. (2012).

Prenylation, which is also called “lipidation,” is the *covalent* addition of *hydrophobic* molecules to a protein or chemical compound (Zhang and Casey, 1996) (Figure 6). The _α_- and _γ_ but not the _β_ G-proteins (Figure 7) are important targets of prenylation-farnesylation. Protein prenylation involves the transfer of either a farnesyl or a geranyl-geranyl moiety to a C-terminal cysteine(s) of the target protein. Three enzymes can carry out prenylation in the cell. They recognize the CaaX box at the C-terminus of the target protein. C is the cysteine that is prenylated. Any protein ending with such a terminus can be prenylated. For figure, see Figure 2 in Shen et al. (2015). Prenylation is an important process to mediate protein–protein interactions and protein-membrane interactions. Through the attachment of a hydrophobic tail a hydrophilic protein can be attached to another protein that is more hydrophobic, or to a membrane. Prenylation is only operational in eukaryotes, not in prokaryotes (Vögler et al., 2008; Wikipedia, 2018e).

A relevant question is what function the attachment of a horseshoe-shaped very flexible hydrophobic tail to a G Protein serves? Is it simply attaching a G protein to a GPCR or to the lipid part of the plasma membrane (Figure 7)? Or, given the horseshoe structure of farnesol, is it more complex?

## From Microbial Rhodopsin’s Retinal to Eukaryotic Farnesyl?

### Cell-Physiological Archeology: An Alternative for Microbial Retinal Was Required for GPCR Functioning in Eukaryotes

Microbial rhodopsins need the chromophore retinal to be functional (§5). In contemporary eukaryotic rhodopsins, retinal is still the chromophore as it was in ancient microbial type-I rhodopsin. In (most) GPCRs it is not. What did change in the course of evolution? In contrast to prokaryotes, eukaryotes cannot synthesize retinal by themselves. They depend upon the metabolization of (dietary) vitamin A into retinol. But this source is insufficient to accommodate all eukaryotes all the time. Hence, an alternative had to be introduced. Apparently farnesyl, which is synthesized in the mevalonate biosynthetic pathway (Figure 2), and which is omnipresent in all eukaryotes, but not in (all) prokaryotes, was an acceptable substitute. Such a functional group should be *correctly positioned* inside a GPCR, from the inside of a cell, not from the extracellular site (e.g., blood). Apparently, G proteins that could be “prenylated” were an option to achieve this. With the exception of rhodopsin, intramolecular inorganic ion transport through a 7 TM-GPCR is not considered as a major component in eukaryotic GPCR functioning. Such passive transport is considered to be the function of canonical ion channels. This is remarkable. Has this important part of the functioning of rhodopsin, the predecessor of numerous GPCRs, been completely lost in evolution? Or is it still in place but in an overlooked *modified* form? If it has indeed been lost, which other mechanism replaced it? Does the answer reside in G proteins, and in the fact that some can be “prenylated”? Does the attachment of a farnesyl-group to a G-protein represent a functional substitute for the “chemical (flip-flop) valve function” of ancient microbial retinal?

### The Horseshoe-Shape and High Flexibility of Both Retinal and Farnesol/FLS: Highly Conserved in Evolution, Thus of Functional Importance

Figure 7 illustrates the contemporary view that a prenyl-farnesyl group is a way to attach the G protein(s) subunits to the membrane or to the GPCR. We ponder whether attachment to the lipid bilayer part of the membrane is the only function of the prenyl-farnesyl group. Why cannot a linear molecule be used instead of a *horseshoe-shaped molecule* such as farnesyl? In case farnesyl inserts itself in between some of the helices of the GPCR, a more complex function has to be envisaged.

A second question: why is the prenyl group *covalently* linked to the G protein? This covalent attachment suggests that the prenyl group needs to be tightly anchored to the G protein in a particular geometry. This may enable it to undergo *controlled* allosteric changes (flip-flopping changes: see next section). In microbial rhodopsins, retinal is not loosely attached either, but *covalently linked* to a lysine residue in the seventh transmembrane domain of the protein.

### Retinal Flip-Flopping: A Flexible Molecular Valve?

**FIGURE 8:**
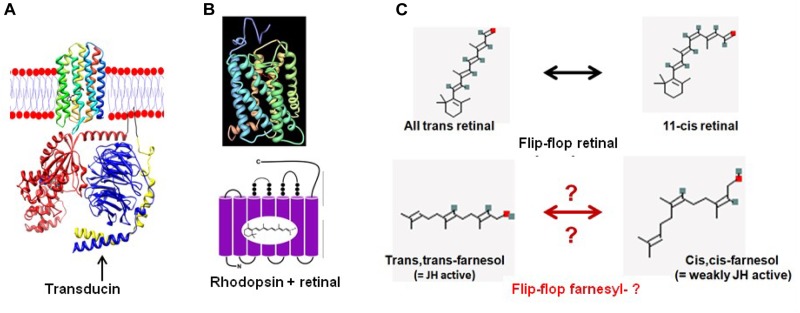
From retinal in microbial rhodopsins to the prenyl-farnesyl group in eukaryotic GPCRs. **(A)** Schematic representation of microbial sensory rhodopsin II embedded in the membrane with transducin under it (both figure and legend borrowed from Wikipedia, 2018f). Rhodopsin is colored in a rainbow with the N-terminus red and the C-terminus blue. Bound retinal on the inside is in black for ease of visualization. For the transducin, the Gt-alpha subunit is red, beta is blue and gamma is yellow. Pseudo anchoring sites in black. The Gt-alpha subunit has a bound GDP that is colored by atom. The protein structures were created using UCSF chimera and then placed together in adobe illustrator. This illustration is the author’s own using the publicly available pdb data. Released into the public domain by the author (Dryan at English Wikipedia). **(B)** Crystal structure of Bovine Rhodopsin A (from Palczewski et al., 2000). Crystal structure of rhodopsin: a G protein-coupled receptor (GPCR). Note: originally uploaded (in 2006) to en:Wikipedia by Roland Deschain. Under: linear representation of the rhodopsin-retinal complex. **(C)** Upon absorption of a photon ( = photo-activation), the 11-*cis*-retinal chromophore isomerizes to the all-*trans* state. Farnesol, which is synthesized in all eukaryotes, occurs in several isoforms. For most of them, the function is largely unknown. We hypothesize that upon binding of a ligand to its GPCR receptor, isomerization of the prenyl group may occur, resulting in a switch toward the receptor’s active conformation. However, this is hypothetical because experimental data for this possibility are currently lacking.

The idea that farnesyl may act as *a flip-flopping hydrophobic molecular valve* inside a GPCR (like a slightly flexible and compressible cork in a wine bottle) may initially look implausible. Yet, it is not at all impossible. Such system was already operational in the ancient microbial rhodopsins. Indeed, isomerization of 11-*cis* retinal, the chromophore in rhodopsins, into all-*trans*-retinal by light sets off a series of conformational changes (‘bleaching’) in the opsin protein, resulting in a form called metarhodopsin II (Meta II). That activates an associated G protein, transducin, to trigger a cyclic guanosine monophosphate (cGMP) second messenger cascade (Figure 8A). Because textbooks tend to depict retinal isomers as linear molecules (Figure 8B,C) rather than their true 3D conformation, the importance of the flip-flopping change, although well-documented (Figure 8C) may have escaped the attention of some researchers. 11-*cis*-retinal has a horseshoe shape while all-*trans*-retinal is straighter. Thus light flips the horseshoe shape into a straighter conformation. The change activates the system, which involves intra-7 TM rhodopsin H^+^-transport. Apparently this flip-flopping causes some “shearing” in the helix bundle with increased passage of H^+^ into this straighter conformation of retinal as a result. In other words: the flip-flopping activity causes tiny leaks of H^+^ through the intramolecular gateway/channel formed by three out of seven transmembrane helices (Kandori, 2015; Kaneko et al., 2017).

### Farnesyl-: Also a Flip-Flopper? A Substitute for Retinal?

To support the argument that a prenyl/farnesyl group may have become a substitute for rhodopsin’s retinal in eukaryotic GPCRs, one has to keep in mind which properties of retinal should be present in a substituting prenyl-group. In short:

**Retinal** is a hydrophobic photosensitive, highly flexible horseshoe-shaped (when in the *cis–cis* isoform) *micro-lipid droplet* that is *covalently* bound and held in a horizontal position inside the 7 TM transmembrane rhodopsin. There it enables the transport of H^+^ by *a flip-flopping change in isomerization* (from *cis–cis*-, to *trans–trans*-) under the influence of light. The molecular size of retinal is 284.443 g/mol, and its rotatable bond count (a measure for its flexibility) is 5.

A **farnesyl-group** is also hydrophobic. It is not documented as being photosensitive. Like retinal, it also has a horseshoe-shape (when in the all-*trans* isoform). It also occurs as a micro-lipid droplet that is covalently bound to an α or γ G-protein that is essential for attachment to a matching GPCR. It is not known whether it is also held in a horizontal position when attached to its binding pocket in the GPCR. The various isoforms found in farnesol extracts (e.g., from plants) suggest that isomerization can occur under natural, physiological conditions. It has not yet been investigated whether or not such isomerization occurs when, e.g., a ligand binds to its GPCR ( = the counterpart of photo-isomerization of retinal). The best documented isomers are: *trans, trans*-farnesol, 2-*cis*,6-*trans*-farnesol, 2-*trans*,6-*cis*-farnesol and *cis–cis*-farnesol (see PubChem). Whether or not a flip-flopping mechanism is at work is currently unknown. The molecular size of farnesol is 222.372 g/mol, and its rotatable bond count is 7.

**Juvenile hormones** are esters of farnesol. No data are available on their possible use in prenylation. Their MW varies from 294.435 g/mol for JH I to 266.381 g/mol for JH III. Their rotatable bond counts are 10 and 6 respectively. According to Wigglesworth (1969), the effects observed in bioassays detecting JH activity by various compounds *are not qualitatively but mainly quantitatively different*. This probably means that the same molecular principle is involved. In our opinion, this principle is: as long as [Ca^2+^]i can be kept low, the juvenile state will prevail (De Loof et al., 2014). Compounds which display JH-hormone activity act at the contact zone between extracellular environment (blood) and the plasma membrane. Prenyl groups, which -in our opinion- also contribute to restricting Ca^2+^ entry into the cytoplasm act from inside the cell. Apparently, the underlying basic mechanism may be the same.

*Farnesyl- has at least some of the right properties needed to act as a substitute for retinal.* It has a similar size, a similar 3D horseshoe conformation, it is hydrophobic and very flexible (rotatable bond count of 7 versus 6 of retinal). It occurs in various isoforms (e.g., in extracts from flowers used in the perfume industry) making a flip-flopping transition under natural conditions at least theoretically possible. In various bioassays for JH differential activities between some of the tested isomers were shown. According to Wigglesworth (1969), who compared 42 farnesol-related compounds using a JH-bioassay, the all-*trans* farnesol form is the most active. This does not necessarily imply that this also holds true for its role in prenylation. The unanswered key question is that although it seems to be theoretically possible, for farnesyl to flip-flop under physiological conditions, e.g., inside a GPCR, does it really happen? And, if it does, is such flip-flopping isomerization somehow linked with the changes in the 3D conformation ( = allosteric change) that take place when a ligand attaches to its matching binding pocket inside a GPCR? To our knowledge, this has never been investigated, probably because the physiological importance of such study was not apparent in the past. No study has been undertaken in the past to compare the 3D conformational changes by various farnesol isomers and their potency of prenylation.

## Model for the Role of Prenylation in GPCR Functioning

### Criteria That the Model Needs to Meet for Validity

A model that tries to explain the mechanism underlying the conformational changes that are essential for GPCR activation has to answer many questions. Several questions in the following list were already designated as “tough” by Gether (2000).

(1) Why do the heterotrimeric G proteins function as dimers (Bastiani and Mendel, 2006)? Why do they have to be positioned very close to the cytoplasmic side of a GPCR as if they function like a drain stopper? Why can’t they be attached, if that is needed at all, e.g., to the peripheral cytoskeleton, at close proximity from the plasma membrane?
(2) If the prenyl group is inserted into the lipid bilayer itself to establish an anchor by hydrophobic interactions, why is the farnesyl-group so small, and why does it have a horseshoe-shape? A much larger prenyl-tail would have more hydrophobic interaction and consequently be a stronger anchor.
(3) Is a prenylated G protein held in place, thus attached to the GPCR, because the prenyl group it carries is inserted in between the helices of a GPCR?
(4) *Or does the opposite situation prevail*: is it the G protein that positions the farnesyl/prenylgroup in such an orientation inside a binding pocket in the GPCR so that *it can act like a valve-like stopper that restricts untimely influx of Ca^2+^* into the cytoplasm?
(5) Why does the isomeric 3D conformation matter so much in some bioassays? Why does a farnesyl-group assumes the *trans–trans* configuration (and retinal in the *cis–cis*) to be active?
(6) Retinal flip-flops upon photoactivation: does a prenyl-group also flip-flop, not upon photo-activation, but upon binding, e.g., of a chemical ligand to the matching GPCR?
(7) The literature on “the downstream effects” of the activation of G-proteins following binding of a ligand to its matching GPCR is extensive. Briefly, there are two principal signal transduction pathways involving the GPCRs: the cAMP signal pathway and the phosphatidylinositol signal pathway (Figure 5). These pathways will not be reviewed here. How does an activated allosteric transition in a GPCR trigger any of the two pathways? Can this be achieved without a flux of (a small amount) Ca^2+^ or/and H^+^ that accompanies the allosteric transition?

### The Farnesyl/Prenyl Flip-Flopping Model

The basic philosophy underlying our model states that if the flip-flopping model of retinal in ancestral microbial rhodopsins functioned flawlessly for so many years – in fact into the current eukaryotic rhodopsins – it would thus be surprising that this successful physiological principle would not have been continued when the novel cell format, the eukaryotic one, appeared on the scene. But because eukaryotes cannot synthesize retinal by themselves in the same way as prokaryotes do, an alternative molecule for retinal was introduced. The eukaryotic mevalonate biosynthetic pathway may have advanced the sesquiterpenoid farnesyl- group as a potential candidate for substituting retinal. To position such group in the right position inside a GPCR, prenylation was used.

In our model, the attachment of a ligand, e.g., a hormone or a neurotransmitter, to its intramolecular binding pocket, may cause a shift in the mutual positions of the helices (usually 3, like in a cycle of the SERCA-Ca^2+^ pump; Vandecaetsbeek et al., 2011) forming a possible transient intramolecular microchannel for selected inorganic ions (in particular H^+^ and Ca^2+^). This short-lived widening of the channel would allow the flux of a small amount of some ions with signaling capacity (Ca^2+^ and/or H^+^) to come into contact with a G protein attached to the cytoplasmic side of the GPCR. Because small changes in concentration of both Ca^2+^ and/or H^+^ can alter the 3D conformation of many types of macromolecules as mentioned before, perhaps, the resulting effect on the G proteins could be the initiation of their subsequent activation by either cAMP or IP3. Whether such changes might involve a change in isomerization of a prenyl group remains to be investigated. The main role of such isomerization might be to close the microchannel promptly.

### Differences With the Classical Models on the Role of Prenylation

Our flip flopping model gives more weight to prenylation than other classical models, including the “consensus model.” It is assumed by others that one of the functions of prenylation or lipidation as it is also called, is attaching G proteins to the plasma membrane and/or to the GPCR with which they form a tandem functional unit. In simple words: prenylation is a system of gluing together molecules by means of a lipid-like glue, bringing them in close proximity to each other.

Our model states on the other hand, that a prenylated G protein may bring the prenyl/farnesyl group to the exact position where it can be inserted into the GPCR. Once it has been inserted and continues to remain covalently anchored, the farnesyl group can then function as a flexible, horseshoe-shaped molecular lipidic valve that, when in the contracted horseshoe-shaped isomeric form (*trans–trans*), minimizes the passage of solutes through the micro-channel present in any GPCR. In simple words: a flexible farnesyl-group may function like a sliding cork that is used for sealing a bottle.

The nature of the allosteric change induced by ligand binding may, again in our opinion, not differ very much from the normal functioning of a normal (canonical) Ca^2+^ channel. Farnesol has been shown to act as a inhibitor of a voltage-gated Ca^2+^ channel-type in a vertebrate arterial system and some cultured cells (Luft et al., 1999; Roullet et al., 1999; De Loof and Schoofs, 2019). Farnesol, in concert with other factors, keeps the different subunits (4, Figure 3) tightly attached to each other that almost no Ca^2+^ can pass through the intramolecular microchannel. When farnesol is no longer continuously present in the extracellular (and the intracellular one as well?) environment, the closed state of the channel is relaxed/lifted, and Ca^2+^ flows through (Roullet et al., 1999). Thus, in its modes of action, farnesol as a (hypothetical) hormone, or as an inbrome, or through farnesyl-prenylation of G proteins, the main effect of its binding to a transmembrane protein is minimizing the untimely influx of excess Ca^2+^.

### Does Our Model Answer the Specificity Question? the “Double Asymmetry Principle” Underlying Differentiation

As Gether (2000) already queried: “How can, for example, so many chemically diverse hormones, neurotransmitters, and other signaling molecules activate receptors believed to share a similar overall tertiary structure?”

This question addresses the basic principle of differentiation during embryonic development of multicellular organisms, animals, plants, etc. Which universal principle underlies differentiation? Like all basic principles in nature, it is rather simple, but despite its simplicity, it allows an endless variability. According to De Loof’s (1993, 2016) hypothesis, with few exceptions not taken into account, it says: “Keep the genome constant during the successive mitotic divisions that occur in a developing embryo, but change, again and again, the ionic and macromolecular environment around the genome (DNA). A means to achieve this is by making use of the universally valid “double asymmetry principle” as outlined by De Loof (1993). All differentiated cells have the same genome, but *they differ primarily in their plasma membrane-cytoskeletal complexes*. The latter are instrumental in controlling gene expression by both inorganic ions and by transcription factors (De Loof, 2016). Thus (hormonal) ligands will only activate their matching receptor in those cell types that express it. Hence, it does not matter much that different ligands use the same downstream signaling systems as long as the receptors present in the different cell types are cell-specific.

## Discussion

Our model primarily focusses on the question whether or not farnesyl- has a key function as a chemical valve in restricting the untimely influx of excess Ca^2+^ (and perhaps of H^+^ as well) in GPCRs to which a prenylated G protein attaches. It has to be seen in a much broader context. Indeed, farnesyl- and farnesol are formed in the mevalonate biosynthetic pathway of all eukaryotes, indicating that this pathway is a key player in cell physiology. In vertebrate physiology, farnesylpyrophosphate is best known as the precursor for the biosynthesis of squalene, which is also the precursor for cholesterol. This precursor function is usually considered to be the key role of farnesyl-PP. This view is questioned because some invertebrates, in particular insects, do not have the gene coding for squalene synthase. Hence they cannot synthesize cholesterol by themselves, but they nevertheless did very well in evolution. In addition, farnesol serves as direct precursor for the synthesis of JH, which are only simple esters of farnesol. Vertebrates do not have these esters, but have farnesol (De Loof et al., 2015). Hence that function too is not an essential key function of the mevalonate pathway.

Its presence in the ancient unicellular ancestors of multicellular eukaryotes (Opisthokonts), in particular of animals, suggests that the key function of the mevalonate pathway must be truly essential for cell physiology (De Loof and Schoofs, 2019). Luft et al. (1999) and Roullet et al. (1999) advanced evidence for such an indispensable function, namely in Ca^2+^-homeostasis, more specifically in keeping [Ca^2+^]i low by inhibiting some types of Ca^2+^ channels. These authors only studied voltage-gated channels present in the plasma membrane. Given the multitude of different Ca^2+^ channels, their structural similarity and their distribution in various membrane-types, e.g., the important homotetrameric Ryanodine receptor(s) (RyRs: ryanodine is a plant alkaloid) in the endoplasmic reticulum, it may be possible that at least some of them also have a binding site for endogenous farnesol-like sesquiterpenoids. No experimental data have yet been reported, but it is known that the transmembrane domain of RyRs represents a chimera of voltage-gated sodium and pH-activated ion channels (Efremov et al., 2015; Zalk et al., 2015). Not only Ca^2+^ channels may have a binding site for endogenous sesquiterpenoids, some Ca^2+^ pumps have it as well. The SERCA Ca^2+^-pump has a binding site for a potent sesquiterpenoid blocker, namely thapsigargin. De Loof (2017) suggested that thapsigargin binds with greater affinity to the binding pocket of the still not yet unequivocally characterized endogenous ligand, which, perhaps, may be the endogenous sesquiterpenoid farnesol.

Given the fact that rising [Ca^2+^]i in the cytoplasm is very toxic and thus has to be removed, the question emerges whether farnesol-like endogenous sesquiterpenoids may act as “guards” that control and limit Ca^2+^ entry and passage, not only at voltage-gated Ca^2+^-channels but in all possible routes along which Ca^2+^ can pass, including routes in both the plasma membrane and in intracellular membranes. In our opinion this might very well be the case. Our major argument is that the Ca^2+^-homeostasis system must act as an integrated system involving many contributing factors all keeping [Ca^2+^]i low in the same direction. Indeed, it would be illogical to inhibit a Ca^2+^ channel, and concurrently transport Ca^2+^ in the opposite direction. Perhaps, the overlooked key function of endogenous farnesol-like sesquiterpenoids as one of the triggers for GPCR activation is to hold together the different helices of all(?) types of Ca^2+^transporting transmembrane molecules by means of “a sticky lipidic stopper.”

With respect to future research, a few remarks. Searching the potency of farnesol and some of its esters, the JHs, it has been shown that the *trans–trans* form is the most active isomer. The *cis–cis* form is much less active. However, which isomeric form is preferentially used in prenylation and under which conditions isomerization occurs is still unknown. Hopefully, in the near future, alternatives for detergents in extraction procedures, which would leave the prenyl group intact, will be developed, so that, e.g., cryo-EM and NMR studies can be used to localize these groups in the GPCR complexes.

In addition to its mere scientific interest, the role of the mevalonate pathway with respect to Ca^2+^ homeostasis and of prenylation in particular may have as yet undervalued effects in, e.g., Alzheimer’s- and other major disorders (Jeong et al., 2018). In our opinion, if one invests efforts in studying the relationship between a given disorder in which Ca^2+^-homeostasis is known to play a role, e.g., Alzheimer (Mattson and Chan, 2001; Mattson, 2012; Jeong et al., 2018) one should give attention to possible shifts in isomers, e.g., from *trans–trans* farnesol which naturally occurs in brain tissue and which is a good blocker of voltage-gated Ca^2+^ channels (Roullet et al., 1999), to other less potent isomers. To date, nothing is known about the presence of the less potent isomers in the healthy- and disease-states. Perhaps, some of the prenylation-dependent disorders have a problematic prenylation flip-flopping system?

Our final conclusion is that farnesol-like endogenous sesquiterpenoids with their very ancient evolutionary origin, deserve to become “noble knowns,” particularly in basic and applied biomedical research.

## Author Contributions

All authors listed have made a substantial, direct and intellectual contribution to the work, and approved it for publication.

## Conflict of Interest Statement

The authors declare that the research was conducted in the absence of any commercial or financial relationships that could be construed as a potential conflict of interest.

## References

[B1] Bargmann C. I. (1998). Neurobiology of the *Caenorhabditis elegans* genome. *Science* 282 2028–2033. 9851919 10.1126/science.282.5396.2028

[B2] Bastiani C. Mendel J. (2006). “Heterotrimeric G proteins in C. elegans,” in *WormBook*. Greenwald I. ed (Pasadena, CA: California Institute of Technology). 10.1895/wormbook.1.75.1 PMC4781550 18050432

[B3] Bellés X. Martin D. Piulachs M. D. (2005). The mevalonate pathway and the synthesis of juvenile hormone in insects. *Annu. Rev. Entomol.* 50 181–199. 15355237 10.1146/annurev.ento.50.071803.130356

[B4] Bondke Persson A. (2013). G – proteins – receptors, signals and function. *Acta Physiol.* 209 91–93. 10.1111/apha.12149 23910385

[B5] Brooks C. Murphy J. Belcastro M. Heller D. Kolandaivelu S. Kisselev O. (2018). Farnesylation of the transducing G protein gamma subunit is a prerequisite for its ciliary targeting in rod photoreceptors. *Front. Mol. Neurosci.* 11:16. 10.3389/fnmol.2018.00016 29410614 PMC5787109

[B6] Caers J. Peymen K. Suetens N. Temmerman L. Janssen T. Schoofs L. (2014). Characterization of G protein-coupled receptors by a fluorescence-based calcium mobilization assay. *J. Vis. Exp.* 89:e51516. 10.3791/51516 25146596 PMC4457351

[B7] Cavalier-Smith T. (2017). Origin of animal multicellularity: precursors, causes, consequencesthe choanoflagellate/sponge transition, neurogenesis and the Cambrian explosion. *Philos. Trans. R Soc. Lond. B Biol. Sci.* 372:20150476 10.1098/rstb.2015.0478 PMC5182410 27994119

[B8] De Loof A. (1993). Differentiation: “Keep the genome constant but change over and over again its ionic and/or macromolecular environment”. A conceptual synthesis. *Belg. J. Zool.* 123 77–91.

[B9] De Loof A. (2015). The essence of female-male physiological dimorphism: differential Ca2+homeostasis enabled by the interplay between farnesol-like endogenous sesquiterpenoids and sex-steroids? The Calcigender paradigm. *Gen. Comp. Endocrinol.* 211 131–146. 10.1016/j.ygcen.2014.12.003 25540913

[B10] De Loof A. (2016). The cell’s self-generated “electrome”: the biophysical essence of the immaterial dimension of Life? *Commun. Integr. Biol.* 9:e1197446. 10.1080/19420889.2016.1197446 27829975 PMC5100658

[B11] De Loof A. (2017). Calcitox-aging counterbalanced by endogenous farnesol-like sesquiterpenoids: an undervalued evolutionarily ancient key signalling pathway. *Commun. Integr. Biol.* 10:e1341024. 10.1080/19420889.2017.1341024 28919940 PMC5595427

[B12] De Loof A. De Haes W. Janssen T. Schoofs L. (2014). The essence of insect metamorphosis and aging: electrical rewiring of cells driven by the principles of juvenile hormone-dependent Ca2+-homeostasis. *Gen. Comp. Endocrinol.* 199 70–85. 24480635 10.1016/j.ygcen.2014.01.009

[B13] De Loof A. Marchal E. Rivera-Perez C. Noriega F. G. Schoofs L. (2015). Farnesol-like endogenous sesquiterpenoids in vertebrates: the probable but overlooked functional “inbrome” anti-aging counterpart of juvenile hormone of insects? *Front. Endocrinol.* 5:222. 10.3389/fendo.2014.00222 25610425 PMC4285131

[B14] De Loof A. Schoofs L. (2019). Mode of action of farnesol, the “Noble Unknown” in particular in Ca2+ homeostasis, and its juvenile hormone-esters in evolutionary retrospect. *Front. Neurosci.* 13:141 10.3389/fnins.2019.00141 PMC6397838 30858798

[B15] Efremov R. G. Leitner A. Aebersold R. Raunser S. (2015). Architecture and conformational switch mechanism of the ryanodine receptor. *Nature* 517 39–43. 10.1038/nature13916 25470059

[B16] Fredriksson R. Schiöth H. B. (2005). The repertoire of G-protein-coupled receptors in fully sequenced genomes. *Mol. Pharmacol.* 67 1414–1425. 15687224 10.1124/mol.104.009001

[B17] Frooninckx L. Temmerman L. Van Sinay E. Beets I. Janssen T. Husson S. J. (2012). Neuropeptide GPCRs in *C. elegans*. *Front. Endocrinol.* 3:167. 10.3389/fendo.2012.00167 23267347 PMC3527849

[B18] Gether U. (2000). Uncovering molecular mechanisms involved in activation of G protein-coupled receptors. *endocr. Rev.* 21 90–113. 10696571 10.1210/edrv.21.1.0390

[B19] Gilman A. G. (1987). G Proteins: transducers of receptor-generated signals. *Ann. Rev. Biochem.* 56 615–649. 3113327 10.1146/annurev.bi.56.070187.003151

[B20] Jeong A. Suazo K. F. Wood W. G. Distefano M. D. Li L. (2018). Isoprenoids and protein prenylation: implications in the pathogenesis and therapeutic intervention of Alzheimer’s disease. *Crit. Rev. Biochem. Mol. Biol.* 53 279–310. 29718780 10.1080/10409238.2018.1458070 PMC6101676

[B21] Kandori H. (2015). Ion-pumping microbial rhodopsins. *Front. Mol. Biosci.* 3:52 10.3389/fmolb.2015.00052 PMC4585134 26442282

[B22] Kaneko A. Inoue K. Kojima K. Kandori H. Sudo Y. (2017). Conversion of microbial rhodopsins: insights into functionally essential elements and rational protein engineering. *Biophys. Rev.* 9 861–876. 10.1007/s12551-017-0335-x 29178082 PMC5711702

[B23] Kang Y. Kuybeda O. de Waal P. W. Mukherjee S. Van Eps N. Dutka P. (2018). Cryo-EM structure of human rhodopsin bound to an inhibitory G protein. *Nature* 558 553–558. 10.1038/s41586-018-0215-y 29899450 PMC8054211

[B24] Kimata N. Reeves P. J. Smith S. O. (2015). Uncovering the triggers for GPCR activation using solid state-state NMR spectroscopy. *J. Magn. Reson.* 253 111–118. 10.1016/j.jmr.2014.12.014 25797010 PMC4391883

[B25] Kowluru A. (2017). Role of G-proteins in islet function in health and diabetes. *Diabetes Obes. Metab.* 19(Suppl. 1), 63–75. 10.1111/dom.13011 28880478 PMC5657296

[B26] Lai D. Wan M. Wu J. Preston-Hurlburt P. Kushwaha R. Grundström T. (2009). Induction of TLR4-target genes entails calcium/calmodulin-dependent regulation of chromatin remodelling. *Proc. Natl. Acad. Sci. U.S.A.* 106 1169–1174. 10.1073/pnas.0811274106 19164553 PMC2633529

[B27] Liang Y. L. Khoshouei M. Deganutti G. Glukhova A. Koole C. Peat T. S. (2017). Phase-plate cryo-EM structure of a class B GPCR-G-protein complex. *Nature* 546 118–123. 10.1038/nature22327 28437792 PMC5832441

[B28] Liang Y. L. Khoshouei M. Deganutti G. Glukhova A. Koole C. Peat T. S. (2018). Cryo-EM structure of the active, Gs protein complexed, human CGRP receptor. *Nature* 561 492–497. 10.1038/s41586-018-0535-y 30209400 PMC6166790

[B29] Luft U. C. Bychkov R. Gollasch M. Gross V. Roullet J. B. McCarron D. A. (1999). Farnesol blocks the L-type Ca2+ channel by targeting the alpha 1C subunit. *Arterioscler. Thromb. Vasc. Biol.* 19 959–966. 10195923 10.1161/01.atv.19.4.959

[B30] Mattson M. P. (2012). Parkinson’s disease: don’t mess with calcium. *J. Clin. Invest.* 122 1195–1198. 22446181 10.1172/JCI62835 PMC3314478

[B31] Mattson M. P. Chan S. L. (2001). Dysregulation of cellular calcium homeostasis in Alzheimer’s disease: bad genes and bad habits. *J. Mol. Neurosci.* 17 205–224. 11816794 10.1385/JMN:17:2:205

[B32] Mertens I. Vandingenen A. Meeusen T. De Loof A. Schoofs L. (2004). Postgenomic characterization of G-protein-coupled receptors. *Pharmacogenomics* 5 657–672. 15335287 10.1517/14622416.5.6.657

[B33] Orrenius S. Zhihotovsky B. Nicotera P. (2003). Regulation of cell death: the calcium-apoptosis link. *Nat. Rev. Cell Biol.* 4 552–565. 12838338 10.1038/nrm1150

[B34] Palczewski K. Kumasaka T. Hori T. Behnke C. A. Motoshima H. Fox B. A. (2000). Crystal structure of rhodopsin: a G protein-coupled receptor. *Science* 289 739–745. 10926528 10.1126/science.289.5480.739

[B35] Ren J. Li X. R. Liu P. C. Cai M. J. Liu W. Wang J. X. (2014). G-protein αq participates in the steroid hormone 20-hydroxyecdysone nongenomic signal transduction. *J. Steroid Biochem. Mol. Biol.* 144(Pt B), 313–323. 10.1016/j.jsbmb.2014.08.006 25125388

[B36] Rogers T. B. Inesi G. Wade R. Lederer W. J. (1995). Use of thapsigargin to study Ca2+ homeostasis in cardiac cells. *Biosci. Rep.* 15 341–349. 8825036 10.1007/BF01788366

[B37] Roullet J. B. Spaetgens R. L. Burlingame T. Feng Z. P. Zamponi G. W. (1999). Modulation of neuronal voltage-gated calcium channels by farnesol. *J. Biol. Chem.* 274 25439–25446. 10464274 10.1074/jbc.274.36.25439

[B38] Safdari H. A. Pandey S. Skukla A. K. Dutta S. (2018). Illuminating GPCR signalling by cryo-EM. *Trends Cell Biol.* 28 591–594. 10.1016/j.tcb.2018.06.002 29945844

[B39] Shen M. Pan P. Li Y. Li D. Yu H. Hou T. (2015). Farnesyltransferase and geranylgeranyl transferase I: structures, mechanism, inhibitors and molecular modelling. *Drug Discov. Today* 20 267–276. 25450772 10.1016/j.drudis.2014.10.002

[B40] Stewart A. Huang J. Fisher R. (2012). RGS Proteins: brakes on the vagus. *Front. Physiol.* 3:95. 10.3389/fphys.2012.00095 22685433 PMC3368389

[B41] Syrovatkina V. Alegre K. O. Dey R. Huang X.-Y. (2016). Regulation, signaling and physiological functions of G-Proteins. *J. Mol. Biol.* 428 3850–3868. 10.1016/j.jmb.2016.08.002 27515397 PMC5023507

[B42] Thal D. M. Glukhova A. Sexton P. M. Chistopoulos A. (2018). Structural insights into G-protein coupled receptor allostery. *Nature* 559 45–53. 10.1038/s41586-018-0259-z 29973731

[B43] Vandecaetsbeek I. Cristensen S. B. Liu H. Van Veldhoven P. P. Waelkens E. Eggermont J. (2011). Thapsigargin affinity purification of intracellular P(2A)- type Ca(2+) ATPases. *Biochim. Biophys. Acta* 1813 1118–1127. 10.1016/j.bbamcr.2010.12.020 21215281

[B44] Vögler O. Barceló J. M. Ribas C. Escribá P. V. (2008). Membrane interactions of G proteins and other related proteins. *Biochim. Biophys. Acta.* 1778 1640–1652. 10.1016/j.bbamem.2008.03.008 18402765

[B45] Wigglesworth V. B. (1969). Chemical structure and juvenile hormone activity: comparative tests on *Rhodnius prolixus*. *J. Insect Physiol.* 15 73–94.

[B46] Wikipedia (2018a). *Calcium Channel*. Available at: http://en.wikipedia.org/wiki/CalciumChannel (accessed November 25, 2018

[B47] Wikipedia (2018b). *Farnesol.* Available at: http://en.wikipedia.org/wiki/Farnesol (accessed November 25, 2018).

[B48] Wikipedia (2018c). *G Protein.* Available at: http://en.wikipedia.org/wiki/Gprotein (accessed November 25, 2018).

[B49] Wikipedia (2018d). *G Protein-Coupled Receptor.*

[B50] Wikipedia (2018e). *Prenylation.* Available at: http://en.wikipedia.org/wiki/Prenylation (accessed November 25, 2018).

[B51] Wikipedia (2018f). *Transducin.* Available at: http://en.wikipedia.org/wiki/Transducin (accessed November 25, 2018).

[B52] Wikipedia (2018g). *Thapsigargin.* Available at: http://en.wikipedia.org/wiki/Thapsigargin (accessed November 25 2018).

[B53] Zalk R. Clarke O. B. des Georges A. Grassucci R. A. Reiken S. Mancia F. (2015). Structure of a mammalian ryanodine receptor. *Nature* 517 44–49. 10.1038/nature13950 25470061 PMC4300236

[B54] Zhang F. L. Casey P. J. (1996). Protein prenylation: molecular mechanisms and functional consequences. *Ann. Rev. Biochem.* 65 241–269. 8811180 10.1146/annurev.bi.65.070196.001325

[B55] Zhang Z. Jin Z. Zhao Y. Zhang Z. Li R. Xiao J. (2014). Systematic study on G-protein couple receptor prototypes: did they really evolve from prokaryo tic genes? *IET Syst. Biol.* 8 154–161. 10.1049/iet-syb.2013.0037 25075528 PMC8687355

